# Ticks infesting humans in Italy and associated pathogens

**DOI:** 10.1186/1756-3305-7-328

**Published:** 2014-07-14

**Authors:** Domenico Otranto, Filipe Dantas-Torres, Alessio Giannelli, Maria Stefania Latrofa, Antonio Cascio, Stefania Cazzin, Silvia Ravagnan, Fabrizio Montarsi, Sergio Aurelio Zanzani, Maria Teresa Manfredi, Gioia Capelli

**Affiliations:** 1 Department of Veterinary Medicine, University of Bari, 70010 Valenzano, Bari, Italy; 2 Department of Immunology, Centro de Pesquisas Aggeu Magalhães (Fiocruz-PE), 50670-420 Recife, Pernambuco, Brazil; 3 Department of Human Pathology, University of Messina, 98125 Messina, Italy; 4 Istituto Zooprofilattico Sperimentale delle Venezie, 35020 Legnaro, Padova, Italy; 5 Department of Veterinary Science and Public Health, University of Milan, 20133 Milan, Italy

**Keywords:** Ticks, Pathogens, Humans, Tick-borne diseases, Italy, Distribution

## Abstract

**Background:**

Ticks may transmit a large variety of pathogens, which cause illnesses in animals and humans, commonly referred to as to tick-borne diseases (TBDs). The incidence of human TBDs in Italy is underestimated because of poor surveillance and the scant amount of studies available.

**Methods:**

Samples (*n* = 561) were collected from humans in four main geographical areas of Italy (i.e., northwestern, northeastern, southern Italy, and Sicily), which represent a variety of environments. After being morphologically identified, ticks were molecularly tested with selected protocols for the presence of pathogens of the genera *Rickettsia*, *Babesia*, *Theileria*, *Candidatus* Neoehrlichia mikurensis, *Borrelia* and *Anaplasma*.

**Results:**

Ticks belonged to 16 species of the genera *Argas*, *Dermacentor*, *Haemaphysalis*, *Hyalomma*, *Ixodes* and *Rhipicephalus*, with *Ixodes ricinus* (59.5%) being the species most frequently retrieved, followed by *Rhipicephalus sanguineus* sensu lato (21.4%). Nymphs were the life stage most frequently retrieved (41%), followed by adult females (34.6%). The overall positivity to any pathogen detected was 18%. Detected microorganisms were *Rickettsia* spp. (17.0%), *Anaplasma phagocytophilum* (0.8%), *Borrelia afzelii* (0.5%), *Borrelia valaisiana* (0.3%), *C.* N. mikurensis (0.5%) and *Babesia venatorum* (0.6%).

**Conclusions:**

Results indicate that people living in the Italian peninsula are at risk of being bitten by different tick species, which may transmit a plethora of TBD causing pathogens and that co-infections may also occur.

## Background

Ticks are amongst the most important arthropod parasites of animals and display a worldwide distribution, being adapted to different environments and climates and host species [1]. Hard ticks (order Ixodida, family Ixodidae) represent the most diverse group, occurring in tropical, temperate and even arctic regions [2]. Their medical and veterinary importance is mostly due to their great capacity of transmitting viral, bacterial, protozoan and helminthic infections to animals, which may cause a diverse range of affections, commonly referred to as tick-borne diseases (TBDs). Importantly, zoonotic TBDs (e.g., anaplasmosis, babesiosis, borreliosis, rickettsioses and tick-borne encephalitis) may be associated with both domestic and wild animals, with a high risk of acquiring infections for humans frequenting tick-infested areas, such as forests, meadow habitats and grasslands [1,3,4].

In spite of the availability of several chemical options for tick control, especially for companion animals and livestock, the incidence of human TBDs is increasing worldwide [1,5]. For example, cases of Lyme borreliosis by *Borrelia burgdorferi* sensu lato are on the rise in the United States, and are related to the occurrence of its competent vector *Ixodes scapularis*. In Europe there is an estimated average of 85,000 cases per year [6]. The development of molecular tools (e.g., reverse-line blotting hybridization, pyrosequencing and next generation sequencing), has refined the current understanding of the distribution of TBDs and the role of certain tick species as vectors of these pathogens [1]. Importantly, the immediate removal of an attached tick, referral to a qualified physician and tick identification in specialized laboratories, is pivotal to prompt the diagnosis and the treatment of any TBDs. In addition, the set-up of surveillance programs is required for understanding the risks of transmission of specific infections in certain areas [1].

With about 40 species [7], the tick fauna in Italy is one of the most diverse across Europe, with more species than countries such as Portugal [8] and the United Kingdom [9,10]. For example, a recent study confirmed a high species richness and abundance of free-living ticks in southern Italy, with up to nine species (i.e., *Dermacentor marginatus*, *Haemaphysalis concinna*, *Haemaphysalis inermis*, *Haemaphysalis parva*, *Haemaphysalis sulcata*, *Hyalomma marginatum*, *Ixodes ricinus*, *Rhipicephalus bursa* and *Rhipicephalus turanicus*) collected in a single forested area [11]. Conversely, the species richness in the environment is lower in central and northern Italy where *I. ricinus* is the most prevalent species [12-14].

The incidence of TBDs in humans in Italy is underestimated because of low rates of notification of cases and scant studies focussing on the detection of pathogens in ticks collected from humans (e.g., ref. [15]). Accordingly, the limited data on ticks, and their associated pathogens infecting humans in Italy may impair the understanding of the risk for TBDs in this country. This aspect is particularly important considering that the Italian peninsula has a great vocation for tourism, mostly during the spring and summer months, which constitute the high-risk period for tick infestation in some areas.

Therefore, the present study aimed to identify the species of ticks removed from humans living in northern, southern and insular Italy, and to molecularly detect associated pathogens.

## Methods

### Tick collection and identification

**Figure 1:**
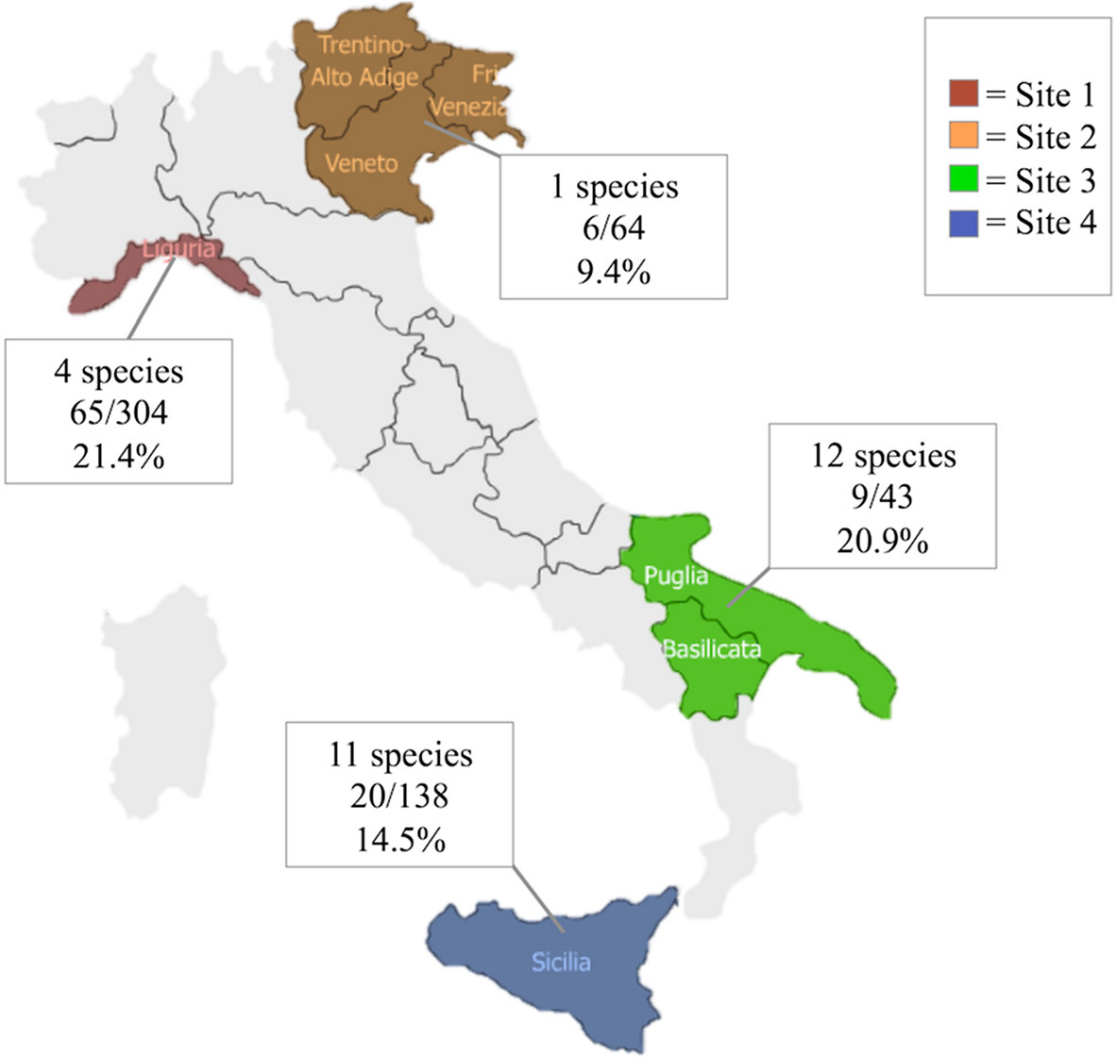
Sampling areas and number of positive specimens collected from each site across the Italian peninsula.

Ticks were collected from humans at different time points, from 1995 to 2011, in northwestern (Savona, Liguria region; site 1), northeastern (Trentino-Alto Adige, Veneto and Friuli Venezia Giulia regions; site 2), southern Italy (Apulia and Basilicata regions; site 3) and Sicily island (site 4) (Figure 1). These sites included four main geographical areas of Italy, representing a variety of natural environments. Most of the inland northern areas are featured by a continental humid climate, whereas the coastal areas of the Liguria region and most of the peninsula are characterized by a Mediterranean temperate climate, with mild winters and hot summers [2,4,7].

All specimens were placed in vials containing 70% ethanol and sent to the laboratories of the Department of Veterinary Medicine of the University of Bari (i.e., ticks from site 3 and 4), University of Milano (i.e., ticks from site 1) and to the Istituto Zooprofilattico Sperimentale delle Venezie (i.e., ticks from site 2). Species identification was performed using taxonomic keys appropriate for each developmental stage (i.e., larvae, nymphs, females, and males) [7,15].

### Amplification of pathogen DNA

All ticks were tested for the presence of pathogens of the genera *Rickettsia*, *Babesia*, *Theileria* as well as of *Candidatus* Neoehrlichia mikurensis. In addition, *Ixodes* ticks were screened for *B. burgdorferi* s.l. and *Anaplasma phagocytophilum*.

DNA was extracted from individual adults, nymphs or larvae, using the All Prep DNA/RNA mini Kit (Qiagen, Inc., Valencia, CA), according to the manufacturer’s instructions, and then frozen at -20°C.

Target genes, primers and probes used, along with the methodologies employed for testing each pathogen, are listed in Table 1. Pathogen identity was confirmed by sequencing each amplicon, using a 16-capillary ABI PRISM 3130 × l Genetic Analyzer (Applied Biosystem, Foster City, CA, USA). Sequence data were assembled and edited with SeqScape software v 2.5 (Applied Biosystem, Foster City, CA, USA), aligned and finally compared with representative sequences available in GenBank.

**Table 1:** **Molecular detection of tick**-**borne pathogens: PCR methods, target genes, primers, nucleotide sequences, amplicon size (base pairs)**

**Species**	**PCR method**	**Target gene**	**Primer**	**Nucleotide sequence (5′-3′)**	**Amplicon size (bp)**	**Reference**
***Anaplasma phagocytophilum***	duplex RT-PCR	msp2	ApMSP2f	ATGGAAGGTAGTGTTGGTTATGGTATT	77	[16]
			ApMSP2r	TTGGTCTTGAAGCGCTCGTA		
			ApMSP2p	TGGTGCCAGGGTTGAGCTTGAGATTG		
	PCR	msp2	msp2-3f	CCAGCGTTTAGCAAGATAAGAG	334	[17]
			msp2-3r	GMCCAGTAACAACATCATAAGC		
***Babesia/Theileria *** **spp.**	RT-PCR Sybr green	18S rRNA	NM-1152as	TTCTACTTTGAACATTTGAAGAATTACTAT	411- 452	[18]
			BJ1	GTCTTGTAATTGGAATGATGG		
			BN2	TAGTTTATGGTTAGGACTACG		
***Borrelia burgdorferi *** **sensu lato**	duplex RT-PCR	23S rRNA	Bb23Sf	CGAGTCTTAAAAGGGCGATTTAGT	75	[16]
			Bb23Sr	GCTTCAGCCTGGCCATAAATAG		
			Bb23Sp	AGATGTGGTAGACCCGAAGCCGAGTG		
	PCR	Flagellin	FLA1	AGAGCAACTTACAGACGAAATTAAT	482	[19]
			FLA2	CAAGTCTATTTTGGAAAGCACCTAA		
***Candidatus *** **Neoehrlichia mikurensis**	TaqMan RT-PCR	groEL	NMikGroEL F2a	CCTTGAAAATATAGCAAGATCAGGTAG	101	[20]
			NMikGroEL R2b	CCACCACGTAACTTATTTAGCACTAAAG		
			NMikGroEL P2a	CCTCTACTAATTATTGCTGAAGATGTAGA		
				AGGTGAAGC		
	PCR	groEL	NM-128 s	AACAGGTGAAACACTAGATAAGTCCAT	1024	[21]
***Rickettsia *** **spp.**	TaqMan RT-PCR	gltA	CS5	GAGAGAAAATTATATCCAAATGTTGAT	146	[22]
			CS6	CATTGTGCCATCCAGCCTACGGT		
			Probe CS5-6	CATTGTGCCATCCAGCCTACGGT		
	PCR	gltA	RpCS.877p	GGGGGCCTGCTCACGGCGG	381	[23]
			RpCS1258n	ATTGCAAAAAGTACAGTGAACA		
	PCR	ompA	Rr190.70p	ATGGCGAATATTTCTCCAAAA	532	[24]
			Rr190.602n	AGTGCAGCATTCGCTCCCCCT		
	PCR	ompB	rompB OFm	GTAACCGGAARTAATCGTTTCGT	489	[25] (modified)
			rompB ORm	GCTTTATAACCAGCTAAACCRCC		

### Statistical analysis

The difference in prevalence of different tick and pathogen species, according to their provenance and tick life stages were tested using the chi-square test and the Fisher’s exact test, when appropriate. GraphPad statistical software (GraphPad Software, Inc.; http://www.graphpad.com/quickcalcs/contingency1/) was used. Differences were considered significant at p < 0.05.

### Ethics statement

Following the patients’ informed consent, ticks were collected by physicians working at the first aid guard and sent, for diagnostic purposes, to the reference laboratories (see above). As this study was based on tick identification and pathogen detection in ticks, thus not requiring any involvement of patients, ethical approval was not needed.

## Results

In total, 561 ticks were identified, including 16 species belonging to the genera *Argas*, *Dermacentor*, *Haemaphysalis*, *Hyalomma*, *Ixodes* and *Rhipicephalus* (Table 2). The tick species most commonly retrieved were *I. ricinus* (59.5%), followed by *Rh. sanguineus* s.l. (21.4%), *H. lusitanicum* (5%) and *D. marginatus* (4.5%) (Table 2). The life stage most frequently collected were nymphs (41%) and females (34.6%), followed by males (13.4%) and larvae (8.4%) (Table 3).

**Table 2:** Number of tick species identified and number and percentage of positivity within each species for one or more pathogens

**Tick species (n)**	No. of positive (%)*
*Argas reflexus* (2)	0
*Dermacentor marginatus* (25)	12 (48.0)^ABC^
*Haemaphysalis inermis* (1)	1
*Haemaphysalis parva* (2)	0
*Haemaphysalis punctata* (2)	0
*Hyalomma detritum detritum* (2)	0
*Hyalomma lusitanicum* (27)	1 (3.7)^Ad^
*Hyalomma marginatum* (2)	0
*Hyalomma* spp. (8)	0
*Ixodes acuminatus* (2)	0
*Ixodes ventalloi* (1)	1
*Ixodes ricinus* (334)	70 (21.0)^Bde^
*Ixodes* spp. (8)	0
*Rhipicephalus bursa* (4)	0
*Rhipicephalus pusillus* (1)	0
*Rhipicephalus sanguineus* s.l. (120)	13 (10.8)^Ce^
*Rhipicephalus* sp. (1)	0
*Rhipicephalus turanicus* (7)	2 (28.6)
Not determined (12)	1 (8.3)
Total (561)	101 (18.0)

(*) Statistically significant differences are marked by equal letters (uppercase letters = p < 0.01; lowercase letters = p < 0.05).

**Table 3:** Number and percentage of tick stages retrieved and their positivity for one or more pathogens

**Life stage**	N° (%)*	**Positive (%)**
Females	194 (34.6)^AB^	35 (18.0)
Males	75 (13.4)^AcD^	18 (24.0)
Larvae	47 (8.4)^BcE^	6 (12.8)
Nymphs	230 (41.0)^DE^	41 (17.8)
Not determined	15 (2.1)	1 (0.6)

(*) Statistically significant differences are marked by equal letters (uppercase letters = p < 0.01; lowercase letters = p < 0.05).

The overall positivity of samples to any pathogen was 18%, with prevalence rates ranging from 9.4% in northeastern Italy to 21.4%, 20.9% and 14.5% in northwestern, southern Italy and Sicily, respectively. The ixodid species most frequently positive for any pathogen was *D. marginatus* (48%), followed by *I. ricinus* (21%), *Rh. sanguineus* s.l. (10.8%) and *H. lusitanicum* (3.7%) (Table 2). Also *Rh. turanicus* ticks were infected (28.6%), but only seven specimens were collected and tested.

A total of 14 microorganism species were identified, most belonging to the genus *Rickettsia* (17.0%), namely *Rickettsia monacensis* (10.1%), *R. massiliae* (2.1%), *R. slovaca* (1.8%), *R. helvetica* (1.4%), and *Rickettsia* spp. (0.5%) (Table 4). Other pathogen species were *A. phagocytophilum* (0.8%), *Borrelia afzelii* (0.5%) and *B. valaisiana* (0.3%), *C.* N. mikurensis (0.5%) and *B. venatorum* (0.6%). The pathogen association in different tick species ranged from two up to eight infectious agents (i.e., in *I. ricinus*). While *Ixodes* spp. harboured *C.* N. mikurensis, *Anaplasma* and *Babesia* spp., *D. marginatus* was the principal carrier of *R. slovaca*, whereas *Rh. sanguineus* s.l. ticks were positive for *R. massiliae*. Co-infections were detected in four *I. ricinus* ticks, namely *A. phagocytophilum/Rickettsia* spp. (1 nymph), *R. monacensis/B. venatorum* (1 nymph, 1 female), and *R. helvetica/B. afzelii* (1 nymph).

**Table 4:** Number and percentage of ticks positive for different pathogen species

**Pathogen (n. of examined ticks)**	**Positive (%)**	**GenBank Accession numbers (gene)**
*Rickettsia* spp. (554)	3 (0.5)	-
*R. monacensis* (554)	56 (10.1)	KJ663734, KJ663735, KJ663744 (*glt*A)
*R. slovaca* (554)	10 (1.8)	KJ663736, KJ663743 (*glt*A), KJ663756 (*omp*B)
*R. helvetica* (554)	8 (1.4)	KJ663739, KJ663745 (*glt*A), KJ663750 (*omp*B)
*R. peacockii* (554)	1 (0.2)	KJ663738(*glt*A), KJ675444 (*omp*A), KJ675443 (*omp*B)
*R. raoulti* (554)	1 (0.2)	KJ663737 (*glt*A), KJ663752 (*omp*B)
*R. aeschilmannii* (554)	2 (0.4)	KJ663742 (*glt*A), KJ663748(*omp*A), KJ663755 (*omp*B)
*R. massiliae* (554)	12 (2.1)	KJ663740, KJ663741 (*glt*A), KJ663746, KJ663747, KJ663749 (*omp*A), KJ663751, KJ663753, KJ663754 (*omp*B)
*R. massiliae/ripicephali* (554)	1 (0.2)	-
**Total Rickettsiae (554)**	**94 (17.0)**	
*Anaplasma phagocytophilum* (372)	3 (0.8)	KJ663729
*Borrelia afzelii* (371)	2 (0.5)	KJ663732
*B. valaisiana* (371)	1 (0.3)	-
*Babesia venatorum* (509)	3 (0.6)	KJ663730
*C.* N. mikurensis (433)	2 (0.5)	KJ663733

Tick-borne pathogens were detected in adult specimens of each ixodid species herein identified, with the exception of *I. ricinus* nymphs and larvae. In particular, *I. ricinus* larvae were infected by *R. monacensis*. Higher tick species variability was found in sites 3 and 4 compared to the northern regions, where, in contrast, ticks carried a larger number of pathogens (Table 5).

**Table 5:** Number and percentage of ticks positive for different microorganism species according their regional distribution

**Region**	**Species (n)**	N (%)*	**Tick-borne microorganisms (n)**
Northwestern Italy	*D. marginatus* (3)	1	*R. monacensis* (1)
(Savona, Liguria)			
	*I. ricinus* (263)	63 (23.9)	*R. monacensis* (54), *A. phagocytophilum* (3), *B. venatorum* (3), *R. helvetica* (3), *B. valaisiana* (1), *Rickettsia* spp. (1), *B. afzelii* (1)
	*Ixodes* spp*.* (7)	0	
	*Rh. sanguineus* s.l. (31)	1 (3.2)	*R. slovaca* (1)
Northeastern Italy (Veneto, Friuli Venezia Giulia and Trentino Alto Adige)	*I. ricinus* (64)	6 (9.4)	*R. helvetica* (4), *C.* N. mikurensis (2), *B. afzelii* (1)
Southern Italy	*A. reflexus* (2)	0	
(Puglia and Basilicata)	*D. marginatus* (12)	7 (58.3)	*R. slovaca* (5), *R. raoulti* (1), *R. peacockii* (1),
	*H. inermis* (1)	1	*Rickettsia* spp.
	*H. parva* (2)	0	
	*H. punctata* (1)	0	
	*Hyalomma* spp. (4)	0	
	*I. acuminatus* (2)	0	
	*I. ricinus* (1)	0	
	*I. ventalloi* (1)	1	*R. helvetica* (1)
	*Rh. sanguineus* s.l. (15)	0	
	*Rh. turanicus* (1)	0	
	*Rhipicephalus* spp. (1)	0	
Sicily	*D. marginatus* (10)	4	*R. slovaca* (4)
	*H. punctata* (1)	0	
	*H. d. detritum* (2)	0	
	*H. lusitanicum* (27)	1 (3.7)	*R. aeschlimannii* (1)
	*H. marginatum* (2)	0	
	*Hyalomma* spp. (4)	0	
	*I. ricinus* (7)	1	*R. monacensis* (1)
	*Rh. bursa* (4)	0	
	*Rh. pusillus* (1)	0	
	*Rh. sanguineus* s.l. (74)	12 (16.7)	*R. massiliae* (11), *Rickettsia* spp. (1)
	*Rh. turanicus* (6)	2	*R. massiliae* (1), *R. massiliae/rhipicephali* (1)

* The prevalence within each tick species is reported only for tick species represented with more than 10 specimens.

## Discussion

Results of this study indicate that people living in the Italian peninsula are at risk of being bitten by different tick species, which may transmit a plethora of microorganisms. The chance of coming into contact with infected ticks is largely dependent upon the environment and the presence of appropriate hosts, and on whether the ticks or the vertebrate hosts act as reservoirs of the infection [26]. In countries like Italy, characterized by a variety of ecological and climatic characteristics, as well as different regional human habits, the distribution pattern of human TBDs varies greatly from north to south [27]. However, the large number of ticks collected from Sicily and northwestern Italy, compared to the other sampling sites, is most likely due to the convenience sample size here examined. For example, finding *I. ricinus* and *Rh. sanguineus* s.l. as the species most frequently detected on humans in northern and southern Italy, respectively, is not surprising. Indeed, *I. ricinus* ticks show a high affiliation to woodland areas of north-eastern and northwestern Italy, where this species finds optimal conditions, in terms of temperature (i.e., 20–23°C) and relative humidity (i.e., 85–98%) for its development [28,29]. Conversely, *Rh. sanguineus* s.l. develops at higher temperatures (e.g., 20–35°C) and variable relative humidity (e.g., 35–95%) [30]. Although the latter species feeds preferentially on dogs, it can also feed readily on people [31]. Moreover, *A. reflexus*, *Rh. turanicus*, *H. lusitanicum*, *H. marginatum* and *D. marginatus* have been reported on humans, displaying an anthropophilic feeding behaviour, in the absence of their main vertebrate host [26,32,33]. In contrast, the occurrence of other ixodids on humans (Table 2) is unlikely. Indeed, differently from hunting ticks (e.g., *Hyalomma* spp.), which actively seek for their hosts in the environment, *Ixodes* and *Haemaphysalis* species adopt an ambush strategy, waiting on a blade of grass, leaves or shrubs for animals to pass by, attaching upon contact with the host [34]. In addition, although some tick species display a strict host specialization, most ticks are generalists [35] and may therefore parasitize a range of different hosts, including humans.

In spite of their small size, the majority of ticks removed in the present study were nymphs (41%), which may be easily overlooked [7,36]. This data is of interest considering that, in other studies [37], only adults were collected from humans and molecularly processed for pathogens. In addition, the detection of *R. monacensis* in *I. ricinus* larvae, not only suggests its vertical transmission, but also indicates their potential role as vectors for this pathogen.

The incidence of human TBDs increased in Italy over the last decade, with 4,604 clinical cases and 33 deaths documented by the Ministry of Health in the period 1998–2002, mainly in southern and insular regions [38,39]. In particular, *R. monacensis* was confirmed to be widespread throughout the country, mainly in association with *I. ricinus*[40]. This bacterium, isolated for the first time in Germany in 2002 [41], is a spotted fever group (SFG) rickettsia, highly prevalent throughout Europe, with rates of infections in *Ixodes* spp. reaching 34.6% in some areas [40]. This pathogen has been detected in ticks from northern Italy [14,42], with a lower estimated prevalence (i.e., 3.7–4.5%) in *I. ricinus* as compared to the present findings. Importantly, the detection of *R. monacensis* in *D. marginatus* represents a new tick-pathogen association. In spite of the large prevalence of this rickettsia throughout Italy, its pathogenicity is still poorly defined, being referred to as a Mediterranean spotted fever (MSF)-like rickettsia [43], inducing fever, general discomfort, headache, joint pain and erythematous rashes at the site of the tick bite.

The detection of *R. massiliae* in *Rh. sanguineus* s.l. and *Rh. turanicus* from Sicily is in line with data from other Mediterranean countries, including Italy, where the pathogen was found in the same tick species [39]. In addition to fever, acute vision loss, pruritic rash, and *tache noire*, *R. massiliae* infection may result in eschars on the scalp and neck and lymphadenopathy. This clinical presentation is similar to that caused by *R. slovaca* and *R. raoultii* infections, which are the most prevalent causative agents of tick-borne rickettsioses in Europe after MSF [44,45]. The finding of *R. slovaca* and *R. raoultii* in *D. marginatus* detached from human patients in southern Italy (i.e., site 3) is consistent with a previous report in ticks from wild boars in Tuscany (central Italy) [46] and, for *R. slovaca* only, in Sicily [47]. The latter confirms the risk posed by this pathogen to humans living on this island, where cases of tick-borne lymphadenopathy syndrome have been recently diagnosed [48]. Finally, *R. slovaca* DNA has been herein amplified for the first time in the only positive specimen of *Rh. sanguineus* s.l. from northwestern Italy. In addition, *R. helvetica* DNA was detected in *I. ricinus*, thus corroborating previous data, reporting a prevalence of the infection in *Ixodes* spp. ranging from 1.5% to 50% [13,14,42]. The presence of this SFG rickettsia in northern Italy has been suspected since 2004, when three cases of a mild form of rickettsiosis were serologically attributed to *R. helvetica*[49]. Moreover, the pathogen has been detected in *Ixodes ventalloi*, a species usually associated with birds in southern Italy, thus raising questions on the role of this tick species as a vector for it [50,51]. Accordingly, birds may play an important role in the maintenance and dissemination of *R. helvetica* in endemic areas [52].

*Rickettsia aeschlimannii*, a species first isolated from *H. marginatum* from Morocco [53], and then in a patient returning to France from the same country with a MSF-like illness [54], has been detected so far in *Hyalomma* ticks worldwide [55]. For the Mediterranean area, this bacterium has been found in *Hyalomma* ticks from Corsica, with an average infection rate of 73.8% [56], as well as in *H. marginatum* from Sardinia [39] and Sicily [47] in 64% and 20.8% of the examined specimens, respectively. Therefore, the detection of *R. aeschlimannii* in a single specimen may be due to the low number of *H. marginatum* specimens collected (*n* = 2). This pathogen was also found in *H. lusitanicum* from Sicily [57]. The pathogenicity of this bacterium to humans remains largely unknown, albeit the onset of MSF-like lesions has been suggested [58].

Surprisingly, *Rickettsia conorii*, the agent of MSF, was not detected in any of the *Rhipicephalus* spp. ticks examined in this study, probably due to the very low prevalence of the infection in nature [59]. Nonetheless, a recent study determined that several pathogenic *Rickettsia* spp. may be much more prevalent than *R. conorii*[60]. For example, *R. massiliae* was more prevalent than *R. conorii* (3.6% vs. 1.3%) in ticks removed from dogs in southern Italy [61] and a retrospective study of 24 human cases of spotted fever from Sicily confirmed infection by *R. conorii* subsp*. israeliensis* in five (20.8%) and *R. conorii* subsp*. conorii* in 19 (79.2%) cases, respectively [62].

Undoubtedly, knowledge on the pathogenic role of rickettsial species increases with the availability of scientific data on their aetiology and distribution. Consequently, the pathogenicity of *R. rhipicephali* detected in *Rh. turanicus* from Sicily is yet to be determined. To the best of our knowledge, *R. peacockii* has been herein reported for the first time in Italy, and for the first time associated with *D. marginatus*, in Europe, as confirmed by the sequencing of three genes. This bacterium, originally found within the interstitial cells of the ovaries and oocytes of *Dermacentor andersoni* in the United States [63], was previously correlated with a reduced prevalence of *R. rickettsii* infection, the agent of Rocky Mountain spotted fever [64]. Remarkably, the presence of *R. peacockii* or a very closely related rickettsia in Europe should be examined with caution. Indeed, although the high genetic similarity between the *R. peacockii* sequences obtained herein and other sequences available in Genbank, the existence of a closely related but different endosymbiont of ticks in Europe cannot be excluded.

Other members of the family Anaplasmataceae were prevalent in ticks herein examined, and therefore may represent a risk to humans throughout the country. For example, results of this study confirm the occurrence of *C.* N. mikurensis and *A. phagocytophilum* in *I. ricinus* from northeastern and northwestern Italy. *Candidatus* Neoehrlichia mikurensis is an obligate intracellular bacterium, isolated for the first time in rats (*Rattus norvegicus*) and in *Ixodes ovatus* ticks from Mikura Island, Japan [65]. However, a retrospective investigation of museum-archived *I. ricinus* female ticks, collected in Moldova in 1960, indicated that this pathogen has remained undetected for rather a long time [66]. Recently, *C.* N. mikurensis has been identified throughout Europe and has been suggested as a causative agent of disease, mainly in immune-compromised patients from Europe [67] and China [68]. Conversely, *A. phagocytophilum* causes granulocytic anaplasmosis in humans and domestic animals, although it may also infect wild animals (e.g., red deer, roe deer, rodents, and hedgehogs), which act as reservoir hosts [69]. Both *C.* N. mikurensis and *A. phagocythophilum* was detected in *I. ricinus* in Italy, with an overall infection rate in ticks of 10.5% and 1.6–19.9%, respectively [14,70-72]. Therefore, results suggest that humans in Italy are most likely exposed to *I. ricinus* ticks infected by *C.* N. mikurensis and *A. phagocytophilum* by frequenting areas where this tick species occurs (e.g. forests, meadows, grasslands, etc.) [14]. Similarly, the risk for transmission of the aforementioned pathogens may also be high in urban parks [69,73], where *I. ricinus* may be brought in by birds [26]. In addition, the role of the Northern white-breasted hedgehog (*Erinaceus romanicus*), living in urban parks of central Europe (e.g., Hungary [74]) should also be considered. Interestingly, these mammals may also serve as major maintenance hosts for *Ixodes hexagonus*[69]. Similarly, the European hedgehog (*Erinaceus europeus*) has been identified as reservoir host for *B. burgdorferi* s.l. genospecies, including *B. afzelii*[75], here detected in *I. ricinus* from northern Italy.

## Conclusions

Data presented here indicate that humans bitten by ticks in Italy are at risk of infection by different pathogens. The occurrence of co-infected ixodids both in nature and in ticks removed from humans indicates that more than one pathogen may be transmitted by the same tick specimen, which may complicate the clinical presentation in humans during multiple infections. Therefore, physicians should be aware of TBDs and of the possible occurrence of atypical clinical presentations, mainly in areas where more than one pathogen has been detected in ticks.

## Competing interests

The authors declare that they have no competing interests.

## Authors’ contributions

DO, GC and FDT conceived and designed the experiments. AC, MTM, GC, DO and SAZ collected tick specimens. FDT, AG, MTM and FM performed the morphological identification. SR, SC, GC and MSL molecularly identified pathogens. DO, GC, AG, FDT, AC, MTM and SR wrote the first draft. All authors read and approved the final manuscript.
